# Neuroinflammation in the Central Nervous System: Exploring the Evolving Influence of Endocannabinoid System

**DOI:** 10.3390/biomedicines11102642

**Published:** 2023-09-26

**Authors:** Sumit S. Rathod, Yogeeta O. Agrawal, Kartik T. Nakhate, M. F. Nagoor Meeran, Shreesh Ojha, Sameer N. Goyal

**Affiliations:** 1 Shri Vile Parle Kelavani Mandal’s Institute of Pharmacy, Dhule 424001, Maharashtra, India; sumit.rathod@svkm.ac.in (S.S.R.); yogeeta.goyal@svkm.ac.in (Y.O.A.); kartik.nakhate@svkm.ac.in (K.T.N.); 2 Department of Pharmacy, R. C. Patel Institute of Pharmaceutical Education and Research, Shirpur 425405, Maharashtra, India; 3 Department of Pharmacology and Therapeutics, College of Medicine and Health Sciences, Abu Dhabi P.O. Box 15551, United Arab Emirates; nagoormeeran1985@uaeu.ac.ae

**Keywords:** neuroinflammation, proinflammatory cytokines, endocannabinoid system, microglia, cannabinoid receptor 1, cannabinoid receptor 2

## Abstract

Neuroinflammation is a complex biological process that typically originates as a protective response in the brain. This inflammatory process is triggered by the release of pro-inflammatory substances like cytokines, prostaglandins, and reactive oxygen and nitrogen species from stimulated endothelial and glial cells, including those with pro-inflammatory functions, in the outer regions. While neuronal inflammation is common in various central nervous system disorders, the specific inflammatory pathways linked with different immune-mediated cell types and the various factors influencing the blood-brain barrier significantly contribute to disease-specific characteristics. The endocannabinoid system consists of cannabinoid receptors, endogenous cannabinoids, and enzymes responsible for synthesizing and metabolizing endocannabinoids. The primary cannabinoid receptor is CB1, predominantly found in specific brain regions such as the brainstem, cerebellum, hippocampus, and cortex. The presence of CB2 receptors in certain brain components, like cultured cerebellar granular cells, Purkinje fibers, and microglia, as well as in the areas like the cerebral cortex, hippocampus, and cerebellum is also evidenced by immunoblotting assays, radioligand binding, and autoradiography studies. Both CB1 and CB2 cannabinoid receptors exhibit noteworthy physiological responses and possess diverse neuromodulatory capabilities. This review primarily aims to outline the distribution of CB1 and CB2 receptors across different brain regions and explore their potential roles in regulating neuroinflammatory processes.

## 1. Introduction

Neuroinflammation is a multifaceted biological framework that normally originates as a brain’s protective response, with the goal of providing safeguarding. Nevertheless, it is possible to develop into a protracted immune system activation, culminating in an unfavorable clinical state [1]. The inflammation of the central nervous system (CNS), which includes the brain and spinal cord, is frequently referred to as neuroinflammation [2]. The expulsion of pro-inflammatory chemicals such as cytokines, prostaglandins, and reactive oxygen and nitrogen species from stimulated endothelium and glial cells triggers neuroinflammatory process. Following this, pro-inflammatory cells from the outer regions enter the CNS [3]. As a consequence, the inflammation of neurons can cause swelling, tissue damage, and neurological dysfunction, as well as acceleration, which leads to memory loss and the onset of neurodegenerative disorders. Hazardous physiological waste products, troublesome self-proteins in autoimmune reactions, the aging process, pathogenic load caused by viruses and bacteria, as well as traumatic neurological and spinal cord lesions, are all common factors for chronic neuroinflammation [4]. Ischemia, neurological diseases (primarily Alzheimer’s disease and Parkinson’s disease) [5], mental health issues (such as major depressive disorder, schizophrenia, and bipolar disorder) [6], neurodevelopmental diseases (such as autism spectrum disorders and epilepsy) [7], and immune-related ailments are all affected by neuroinflammation [8,9,10].

Although the inflammation of neurons is common in many CNS disorders, the distinct inflammatory processes associated with different immune-mediated cell types and the variety of fundamental and operational circumstances affecting the barrier between the blood and the brain are significantly disease-specific attributes. These elements are critical in describing the nature of the condition and directing its treatment strategy [11]. Neuroinflammation comprises a wide range of diverse biological elements, particularly microglia, the endogenous immune cells of CNS; and mast cells, which are critical in regulating immune responses that are both innate and adaptive. In contrast, oligodendrocytes and astrocytes are complex entities that provide architectural and insulative support to CNS axons while also interacting with other immunological components to influence the pro-inflammatory environment. The most important glial community in neurological inflammation acts notably as a component of the blood-brain barrier and plays a crucial role in neural rehabilitation.

The mast cells and microglia are the principal neuro-immune guardians in brain tissue, and they work along with astrocytes to link distal immunological signaling with the CNS throughout the inflammatory process. Each of these cells is capable of recognizing damaging inputs and responding by generating pro-inflammatory cytokines and chemokines; they play crucial functions in a variety of conditions [12]. Microglial cells become more susceptible to external triggers as people age, perhaps encouraging chronic, moderate inflammatory processes that may not diminish with time. This condition was recently dubbed “inflammaging.” [13,14]. The endocannabinoid system (ECS) and its components play a critical role in natural responses to neurological inflammation, brain trauma, and neurodegenerative conditions. It includes the receptors for cannabinoid CB1 and CB2, as well as its main naturally occurring ligands 2-arachidonylglycerol (2-AG) and arachidonoylethanolamine (AEA or anandamide), along with a variety of enzymes involved in their biosynthesis and decomposition [15,16,17]. The current approach expands on this foundation by including compounds that lack a strong affinity for binding for CB1/2 receptors but exhibit cannabimimetic features, successfully directing the control of genuine endogenous cannabinoids effects.

In addition to this, N-acyl ethanolamines (AEA congeners) and lipoamino acidic substances, as well as acyl conjugates of brain chemicals, confer their physiological impacts via different receptor avenues, particularly the peroxisome proliferator-activated receptor (PPAR)-α [18]. The involvement of transient receptor potential vanilloid 1 (TRPV1) and non-cannabinoid G-protein coupled receptors (GPCRs) is integrated with true naturally occurring endogenous cannabinoids, including their biological targets, complicated interconnections, and chemical reactions. All of these components combine to produce the endocannabinoid domain, which is important in coordinating a physiological response to harmful stressors both inside and outside the body.

## 2. Methods

Several electronic search engines were employed to perform the literature review for the current manuscript, such as Science Direct, PubMed, and Google Scholar. Consequently, the keywords for the literature search are neuroinflammation, endocannabinoids, microglia, astrocytes, neuronal circuitry, presynaptic, postsynaptic transmission, gene expression, CB1 receptor, CB2 receptor, proinflammatory cytokines, neuronal physiology, and neurotransmitters.

## 3. Cannabis

Cannabis-based compounds were originally defined as a set of closely associated terpenoids found only in marijuana plants, particularly delta-9-tetrahydrocannabinol (∆9-THC), serving as the principal active constituent [19]. Nonetheless, this expression has come to refer to any chemical that can trigger cannabinoid receptors. Cannabinoid agonists elicit several unique effects in animals such as thermal regulation, analgesic alleviation, reduced physical activity, and immobilized stiffness [20]. In terms of human instances, the most recognizable psychotropic result, especially in connection to cannabis plant application, is a state of moderate euphoria, sometimes described as a “high” [21,22]. However, cannabis produces a wide range of other effects, including increased sensory awareness, faster heart rates, decreased sensitivity to pain, difficulty in concentrating, nausea relief, and increased hunger. It also causes impairments in linear mental tasks, the retention of information, and neurological and mental synchronization. Previous comprehensive data provide further in-depth evaluations regarding these impacts [21,22,23,24].

The species *Cannabis sativa*, commonly referred to as hemp, has a long history because it is one of the oldest and frequently used drugs around the globe [25,26]. The main psychotropic component found in cannabis plants is ∆9-THC [27]. ∆9-THC, like many naturally generated and synthetic cannabinoids, has detectable impacts on motor skills, mental abilities, and sensations of pain [21,28]. Initial investigations demonstrating cannabinoid-like properties in delta-9-hydroxyhexahydrocannabinol (∆9-HHC), the hydrogenated derivative of ∆9-THC [29,30,31] worked as an impetus for the development of a variety of unique cannabinoids, with the goal of exploring their possible therapeutic applications [32]. Cannabinoids of synthetic origin have physical characteristics comparable to their natural equivalents, eliciting a variety of behavioral and biological responses akin to ∆9-THC, but with intensity levels increased by an amount of 5 to 1000, and exhibiting considerable enantioselectivity [21,32]. *Cannabis sativa*, the biological component under consideration, has been used as a form of medicine for centuries in many civilizations [32]. Cannabis first became popular as a medicinal approach five centuries ago in ancient China, during which it was prescribed for ailments such as malaria, diarrhea, and arthritic diseases. It was additionally employed as a postoperative analgesic when combined with wine [33]. Approximately a century before the common period, the plant evolved as a versatile asset, acting as an intoxicating and tranquilizing agent, and aiding in the treatment of diseases characterized by stress, schizophrenia, and psychosis [34].

In a similar line, the Assyrians used cannabis smoking to ease the symptoms of depressed conditions [35]. Between 50 to 70 AD, the Greek physician Pedacius Dioscorides classified a variety of plants, notably *Cannabis sativa*, and explained its value in De Materia Medica. Cannabis did not enter Western healthcare practices till the end of the eighteenth century, owing to its recognized medicinal, anti-inflammatory, anti-emetic, and anticonvulsant abilities [36]. Extracts from cannabis were first used in medical therapies for schizophrenia at the beginning of the twentieth century, most notably as tranquilizers and sedatives [35]. Nevertheless, the ensuing age, notably around the 1930s, saw a significant fall in cannabis’s medicinal applicability [32]. The reason for this drop was its designation as an illegal drug, which imposed tight constraints on its use in the field of psychotherapy. However, after the understanding of marijuana’s major components and the discovery of how the ECS has the ability to control multiple systems inside mental health conditions, there has been a renewed fascination with the use of cannabinoids [37]. Endocannabinoids are lipid molecules that are formed as a result of the metabolic decomposition of lipids [38].

AEA or anandamide, and 2-arachidonoyl glycerol (2-AG) are two substances that have received a lot of attention [39]. AEA and 2-AG are lipids that are neutral and belong to the fatty acid amide (FAA), monoacylglycerol (MAG) groups, etc. Such lipids are synthesized from the phospholipids found in the membranes of cells and which quickly escape [39]. The creation and breakdown of these naturally occurring substances are tightly controlled by sophisticated enzymatic procedures. A number of mechanisms were proposed for controlling AEA fabrication, such as the decomposition of N-acylphosphatidylethanolamine (NAPE) by the metabolic enzyme NAPE-selective phospholipase D, and the collaborative occupation of α, β-hydrolase domain-comprising 4 and glycerophosphodiesterase 1 on NAPE precursors [40]. The progressive destruction of 2-AG inside diacylglycerol (DAG) phospholipids in the membrane results in the synthesis of 2-AG. Phospholipase C (PLC) and either diacylglycerol lipase (DAGL) or diacylglycerol lipase (DAGL) enzymes control this mechanism [41]. At this point, AEA and 2-AG are degraded by enzymes called fatty acid amide hydrolase (FAAH) and monoacylglycerol lipase (MAGL), respectively. The aforementioned enzymes catalyze hydrolysis, yielding arachidonic acid and either ethanolamine or glycerol [41].

Endogenous cannabinoids primarily exhibit their psychoactive properties via two distinct receptor subtypes: CB1 and CB2 [42]. CB1 receptors have been identified in high concentrations in certain brain areas such as the base of the brain, cerebellum, hippocampus, and cortex [42]. In contrast, there is a reduced representation detected in other locations, such as the cerebral cortex, which houses the control centers for respiration and cardiac activity [43]. These types of receptors were additionally identified in nearby tissues such as the intestinal tract, liver, adipose tissue, and immune system cells [44]. The CB1 receptor is a GPCR having seven transmembrane regions as well as internal close to the C- and the N-terminal regions [42]. Presynaptic CB1 receptor stimulation suppresses the release of neurotransmitters inside brain cells, particularly when CB1 receptor activity is high. The association of receptors that are connected with inhibitory G proteins and cannabinoid ligands led to the blockage of adenylyl cyclase and channels containing calcium N and P/Q, in addition to the amplification of the potassium channels and the stimulation of MAP kinase enzymes [45,46]. When the CB1 receptor is activated in the liver, which is generally present at a reduced level, it increases the production of acetyl-CoA carboxylase-1 and fatty acid synthase, causing a rise in lipid synthesis [47]. CB2 receptors, on the other hand, are receptor-linked with G proteins that are largely found in the spleen, tonsils, and immune system cells [48,49]. Their existence in the CNS was recently found, comprising microglial and neural cells [50,51,52]. CB2 receptor seems to have several immunosuppression impacts, including the suppression of proinflammatory production of cytokines [53,54].

In addition, both AEA and 2-AG have non-canonical cannabinoid receptor sensitivity. AEA preferentially activates the TRPV1 route, resulting in an increase in intracellular calcium levels similar to CB1 and CB2 receptor stimulation [55,56,57]. In addition, high AEA levels trigger nuclear receptor peroxisome proliferator-activated receptor gamma (PPARγ) [58]. PPARs are a kind of gene transcription factor that can be stimulated by ligands that belong to the nuclear receptor superfamily, which includes corticosteroid and thyroid-related hormone ligands [59]. PPARs operate as transcriptional controllers, regulating the activation of several genes and altering levels of glucose, metabolism of lipids, vasculature tightening, and responses to inflammation [60]. The stimulation of the PPAR-γ improves insulin sensitivity, suppresses inflammatory processes, and lowers free fatty acid levels in the bloodstream, hindering the start of atherogenesis, promoting an enhanced function of endothelial cells, and decreasing the frequency of cardiac events [60,61].

## 4. Cannabinoid Locations in the CNS

Over the course of history, cannabis has been used to serve recreational and therapeutic uses. New findings have provided light on the effects of cannabis and its constituents (cannabinoids) on the brain and other regions of the CNS. These psychoactive substances have shown potential in the treatment of neurodegenerative and mental disorders associated with neural inflammation, neurodegeneration, and pain [62]. Naturally occurring cannabinoids derived from plants, produced in a laboratory, their target receptors, and the enzymes responsible for their generation and degradation comprise the cannabinoid system [63]. Modifications in the cannabis system are connected with variations in concurrent effects and are linked to the inflammatory manifestations of these diseases [64]. Non-neuronal cells within the CNS have a particular connection with the onset, perseverance, and alleviation of neurological inflammation, neurological damage, and pain conditions [62,65,66]. This in-depth examination focuses on the locations of receptors that recognize cannabinoids and the ECS in different parts of the brain and the CNS, as well as their potential involvement in neurological modulation [67].

**Figure 1:**
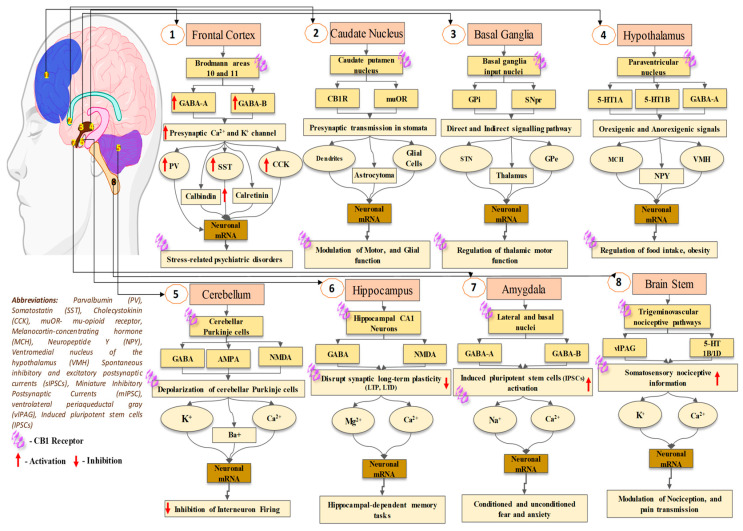
Location of CB1 receptor in the brain, and its physiological retorts.

The prior genomic and pharmacological study has suggested that there are two distinct kinds of GPCR (CB1 and CB2) that are broadly dispersed throughout the human body. CB1A, an alternative variation of the CB1 receptor, has been effectively found in the brain (Figure 1) [68]. This CB1A variation has all of the CB1 receptor’s characteristics but with a somewhat reduced effect [69]. However, the practical importance of this splice variation is unknown due to a lack of supporting data for its self-expression, as other groups of investigators have noticed. Although there is no prior proof for CB2 receptor binding, protein presence, or mRNA expression in the brain [70], certain functional findings do imply the existence of CB2 receptors in cultured cerebellar granular cells with their involvement in ERK pathways, which will lead to increasing the rate of transcription of mRNA in the granular cells [71]. The science of cannabis pharmacology has advanced significantly, culminating in the creation of a variety of specific agonists and antagonists suited for these different receptor subtypes [67,72].

### 4.1. Establishment of the Cannabinoid Receptor 1 (CB1) in Neuronal Tissues

CB1 receptors have a distinct CNS distribution and are found at greater levels in the mammalian brain compared to other recognized GPCRs. CB1 receptor distribution trends have mostly been examined using radioligand binding and autoradiography, as well as immunohistochemistry approaches. Among the most extensively utilized tritiated cannabinoid receptor ligands in affinity trials or for autoradiography are the CB1-selective [3H]SR141716A, [3H]CP55940, [3H]WIN55212-2, and [3H]HU-243. The last three agonists interact with CB1 and CB2 receptors equally strongly. Cannabinoid receptor abundance in the brain rivals that of glutamate [73] and GABA [74]. Cannabinoid receptor levels throughout certain areas are equivalent to dopamine receptor levels and much above neuropeptide receptor numbers. The researchers discovered the highest concentrations of binding in the globus pallidus and substantia nigra pars reticulata, as well as the molecular strata of the cerebellum and the dentate gyrus, which are located inside the hippocampus, using automated technology densitometric analyses on film autoradiographs taken from canine, guinea pig, rat, monkey, and human brain samples [75]. Previously reported data suggested that additional regions of the hippocampus structure, the cortex of the brain, and the striatum display significant binding.

Radioligand affinity is modest in the hypothalamus, basal ganglia, solitary tract nucleus, and spinal cord, with slight inter-species differences [75]. Earlier immunocytochemical assays have demonstrated that the arrangement pattern of the CB1 receptors closely matches that of cannabinoid sites of binding, as discovered by earlier radioligand experiments. According to numerous research [76,77,78,79], these receptors have significantly higher levels of activity in certain locations, including the sense of smell, hippocampus, neocortex, and brainstem. The different arrangement of CB1 receptors in these locations correlates to the regions that are responsible for the different effects of cannabinoid agonists, such as motor control, body position, hypothermia, memory and learning, hunger, vomiting, and alleviation of pain. This finding lends strong credence to the hypothesis that cannabinoids’ activities in the cerebral cortex are primarily mediated by CB1 receptors. CB1 receptor positivity is primarily associated with nerve fiber networks and axon junctions when evaluated at the cellular level, with little activity in neurons and cell nuclei. The above distribution pattern corresponds to the idea of a presynaptic mode of activity.

The positivity of axonal CB1 receptors has been associated with presynaptic nerve terminals of GABAergic neurons, notably ones that are part of cholecystokinin-containing basket cells, in the thoroughly investigated rat hippocampus [80,81,82]. Although there is little proof [76] as well as contradictory views [77,80,83] regarding the existence of CB1 receptors on glutamatergic cells, in situ hybridization investigations suggest that they exist, although in far lower amounts than GABAergic neurons [84]. Antibodies addressing extrinsic regions of CB1 receptors were used to assess their distribution on cell surfaces, allowing the analysis of their outermost expression inside living cells. Additionally, the immunolabeling of the surface of cells’ CB1 receptors in hippocampal nerve cells is particularly numerous at GABAergic synaptic terminals, but no labeling of glutamatergic neurons as a whole is observed [85] (Figure 1).

### 4.2. Cannabinoid Receptor 1 (CB1) in the Communication of Neuronal Signaling Pathways

The reduction in cyclic AMP (cAMP) synthesis induced by agonists [86] and the inhibition of Ca^2+^ inward flows mediated by N- and P/Q-type Ca^2+^ channels [87] are two often documented cytoplasmic effects of CB1 receptor activation in neural networks. These characteristics are similar to those seen in other GPCRs that govern synaptic information transmission (like mGlu, muscarinic, and opioid receptors). Furthermore, these Ca^2+^ channels are mostly found strategically and are required to release neurotransmitters in anticipation of inputs. Cannabis-based compounds also have electrophysiology impacts, such as activating A-type inwardly conducting gates [88] and inhibiting M-type K^+^ channels [89]. Specific effects on K^+^ channels may be caused by variations in cAMP levels [86,88]. Pre-conditioning cells with pertussis toxin inhibit the effect of cannabis on ion channels, showing the presence of Gi/o proteins. Nevertheless, pertussis toxin treatment reveals a cannabinoid receptor-triggered enhancive impact on cAMP buildup inside cultured striatal neuronal cells, revealing the commencement of a new signaling cascade incorporating a Gs protein [90].

Cannabinoids stimulate intrinsic signaling networks in the CNS, including the mitogen-activated protein (MAP) kinase pathway [91], and maybe the c-Jun N-terminal kinase via a phosphoinositide 3’-kinase-dependent process [92]. In addition, earlier research has shown that CB1 receptors can interact with phospholipase C in cerebellar granule cells, causing intracellular calcium to be released [93]. The CB1 receptor, like many other GPCRs, has a reduced response after being exposed to agonists, a condition known as agonist-induced desensitization [94,95,96,97]. This method involves G-protein disengagement after modification by G-protein-coupled receptor kinases (GRKs), resulting in receptor internalization [96]. The phosphorylation of receptors by GRKs causes beta-arrestin binding, which isolates receptors from heterotrimeric G-proteins and drives CB1 receptors into clathrin-coated vesicles [95,96]. CB1 receptor internalization has been examined with transgenic Chinese hamster ovary or AtT20 cell lines [94,95], and native receptors generated in cultured neurons in hippocampal regions or F11 cells [97].

### 4.3. Role of Cannabinoid Receptor 1 (CB1) in the Modulation of Neuronal Physiology

Cannabinoid impacts can be divided into two categories: those influenced primarily by cannabinoid receptors and those impacted indirectly by competing mechanisms [95]. Cannabinoid CB1 receptor stimulus influences the release of many neurotransmitters inside the CNS, including major neurotransmitters that are excitatory and inhibitory like glutamate and GABA [98]. Cannabinoids also influence the discharge of slower-acting neurotransmitters and neurological modulators like opiates, acetylcholine, dopamine, and noradrenaline [99,100,101,102,103,104]. The processes that are driving these consequences at the cellular level are mostly linked to presynaptic Ca^2+^ and K^+^ channel regulation. Cannabinoids modulate GABA transmission in various CNS areas, notably the cortex, the basal ganglia, the cerebellum, and the hypothalamus. However, research has demonstrated that ∆9-THC has an inhibitory impact on GABA absorption in the cerebral cortex and basal ganglia [105,106], and electrophysiology investigations on GABAergic transmission through synapses do not support this influence.

Morphological studies indicate a close connection between high amounts of CB1 receptor immunoreactivity and mRNA and GABAergic nerve cells [77,82,107]. Several neurochemical research [82] and electrophysiology testing [81,108,109] in the hippocampus have demonstrated cannabis’ ability to modulate GABA input from a particular group of inhibitory neurons. Previously reported studies explored that cannabis-based compounds inhibit GABA emission via a presynaptic process in the regions of basal ganglia [110,111,112,113,114], brainstem [115,116], and cerebellum [117,118]. The bulk of data suggest that cannabis regulates GABA distribution mostly through blocking synaptic Ca^2+^ channels (particularly N or N/PQ channels) [101,109], while in other cases, they may directly impact the releasing pathway [85,101,116]. Cannabinoids additionally affect glutamate transport via many crucial mechanisms. Previous investigations have shown that CB1 receptors were present in excitatory synapses of cultured cerebellar granule cells [119].

**Figure 2:**
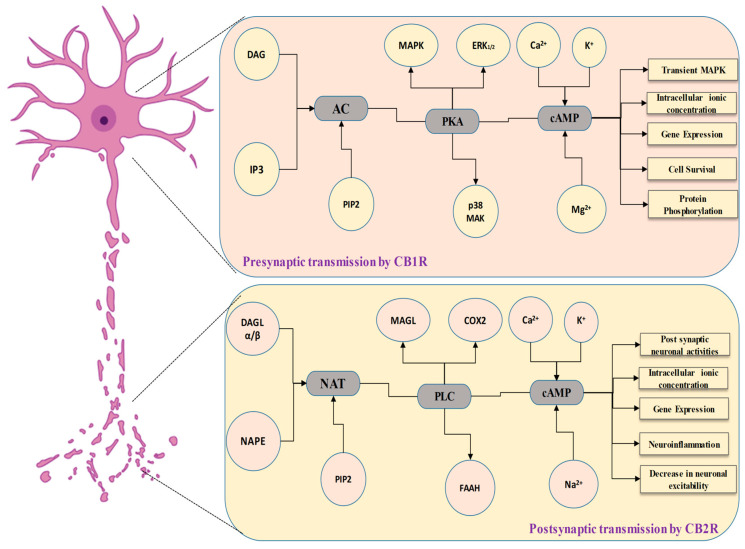
Synaptic transmission by CB1 and CB2 receptors in the neurons, and its physiological retorts.

Cannabinoid receptor activation inhibits glutamate release at the junction among Purkinje cells and longitudinal fibers in cerebellar slices, according to substantial electrophysiological data [120,121,122]. Also, CB1 receptor stimulation inhibits glutamate release via the cerebral nerve in the striatum [123]. In addition, earlier reported studies explored that cannabinoids decrease the glutamatergic transmission of synapses in the middle brain region specifically from periaqueductal grey [116]. Studies also suggested that cannabis-based compounds could impact the discharge of glutamate from a glutamatergic cerebral afferent nerve in the nucleus accumbens, incorporating terminal K^+^ channels [124]. There is uncertainty about excitatory synaptic communication in the hippocampus, with some studies indicating inhibition by cannabis [125,126] and others indicating no impact [105,127] (Figure 2).

### 4.4. The Involvement of the Cannabinoid Receptor 1 (CB1) in Synaptic Modulation

Cannabinoids impede multiple types of plasticity of synapses in the hippocampus, including long-term potentiation (LTP) [126,128,129] and long-term depression (LTD) [126], via a CB1 receptor-dependent process. Studies also evidence that cannabinoids inhibit synaptic plasticity in the brain [130] and striatum [131]. The particular mechanism beyond such impacts is yet unknown. Previous data also suggested that cannabinoids modulate hippocampus neural plasticity by decreasing the release of glutamate [126]. This blockage can be overcome as the neuronal development of NMDA receptors rises, as demonstrated in Mg^2+^-depleted circumstances, or in cases where the postsynaptic cell membrane depolarizes via LTP or LTD induction methods [127].

Because cannabis influences just a specific population of interneurons, the delicate relationship between synaptic plasticity and its effect in inhibiting the transmission of synaptic information is expected to be subtle. This nuance might explain why, contrary to the expected result of a general decrease in inhibitory signaling, cannabis does not increase the plasticity of synapses. The effect of cannabis in inhibiting synaptic communication has the potential to impact the intensity of network oscillations, which are important for activities like memory and learning [81]. According to studies conducted on mice with deficient CB1 receptors, endocannabinoids have a consistent inhibitory effect on synaptic remodeling. This suggests that anandamide may work as an intrinsic messenger, controlling cognitive-linked activities.

### 4.5. Cannabinoid Receptor 1 (CB1) Gene Expression in the Brain

CB1 receptors are the possible therapeutic targets in a variety of illnesses. They nevertheless contribute to the euphoric properties of marijuana, as well as weakening motor abilities and cognition. Such features limit its therapeutic application, prompting a further investigation to uncover the cellular mechanisms driving such cannabinoid-associated behaviors [132]. Understanding these pathways is critical for developing measures to mitigate such unfavorable effects. Adaptation to the recurrent treatment of cannabis agonists is being linked to CB1 receptor desensitization and decreased levels (receptor ablation). Differences in CB1 receptor desensitization and decreased levels have been discovered in rats exposed to THC or synthetic cannabinoids [133]. Identical regional differences in CB1 receptor-reduced levels were identified in the human cerebral cortex [134,135].

Additionally, investigations also demonstrated the presence of CB1 receptors across the brain regions, with significant levels in the hippocampus, cerebral cortex, and cerebellar molecular layers. The hypothalamus, periaqueductal grey, and basolateral amygdala have fewer degrees of expression. The aforementioned distribution pattern corresponds to the cannabinoids’ immediate consequences, including antinociception, catalepsy, hyperlocomotion, hypothermia, and cognitive impairment [136,137]. A line of research has revealed that transcription factors that may be triggered under particular situations naturally exist in the brain and exhibit species-specific changes in activity. Although there has not been an immediate comparison of basal CB1 receptors expression, a more indirect assessment may be conducted, utilizing the BrainStars (B*) database, which comprises DNA microarray information collected from the brain of a mouse [138].

In addition, reverse transcription polymerase chain reaction (RT-PCR) has been employed to evaluate the expression of messenger RNA (mRNA) that encodes the cannabinoid CB1 receptor. The investigation included the post-mortem brain tissue from people who did not have neurological conditions as well as persons who died from Parkinson’s disease or related conditions [136]. CB1 receptor mRNA was found in the striatum as well as extra striatal areas (such as the area of the globus pallidus and substantia nigra). CB1 receptor mRNA levels were shown to be lower in affected neurons in the caudate nucleus, anterior dorsal putamen, and external section of the globus pallidus. However, no significant changes in CB1 receptor mRNA levels were noted in the other examined brain regions. The results suggest that alterations in CB1 receptor mRNA expression developed in Parkinson’s disease; nevertheless, the impact of treatment cannot be ignored. Furthermore, these findings show a relationship between changes in dopaminergic pathways and changes in CB1 receptor mRNA activity [138].

### 4.6. Establishment of the Cannabinoid Receptor 2 (CB2) in Neuronal Tissues

According to investigations in the field of neurological conditions, CB2 principally works in immunological regulation. Brain tissue data from disorders such as Alzheimer’s, Multiple Sclerosis, and ALS show a distinct and particular existence of CB2 in microglial cells [139]. A separate investigation found that CB2 inhibits the production of pro-inflammatory cytokines via stimulated microglia in Alzheimer’s disease [140]. In addition, CB2 stimulation, like CB1, promotes the creation of new synapses in adults, and there is accumulating proof suggesting it may be involved in modulating the permeation of the BBB [141,142]. CB2 receptors were considered to be lacking in the brain in the early stages due to the failure to identify CB2 mRNA transcripts using different methods [143,144,145,146]. These observations contributed to the designation of CB2 receptors as “peripheral” cannabinoid receptors [147,148]. Nevertheless, the theory has been challenged by the discovery of CB2 receptors across the CNS [143,147]. CB2 receptors in the brain differ from CB1 receptors in that they express themselves at a lesser degree than CB1 receptors, hinting that CB2 receptors may not be linked to cannabis-related effects in normal physiological circumstances.

**Figure 3:**
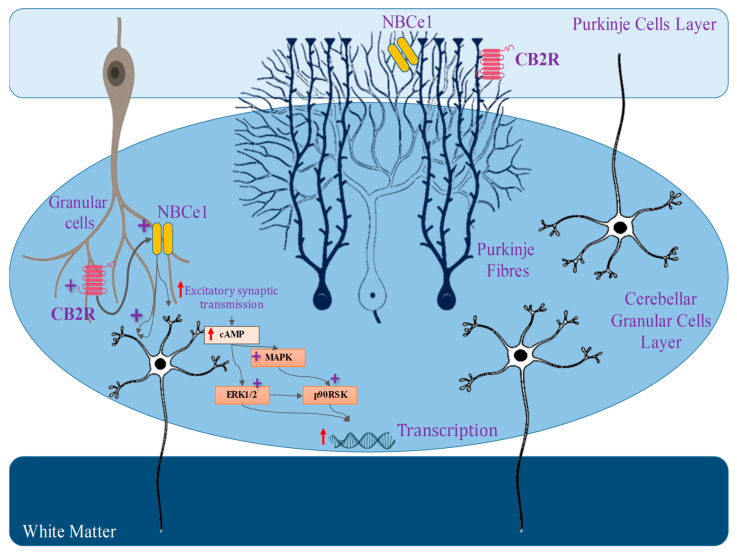
Location of CB2 receptor in the brain, and its physiological retorts.

Because CB2 receptors activity in the brain is very susceptible to induction, there is a fast rise in CB2 receptors activity inside the brain in some clinical settings (including addiction, inflammation, and anxiety) [149]. The evidence shows a link between CB2 receptors regulation and the incidence of many mental and neurological conditions. CB2 receptors are distributed differently in the brain. Because they are mostly present within postsynaptic neuronal somatodendritic areas, triggering them may have different consequences than CB1 receptors [150]. Despite this, CB1 receptors, for example, are mostly found on nerve terminals, namely presynaptic GABAergic endings and dopamine neurons of ventral tegmental area (VTA) [151]. Whenever CB1 receptors are active, they reduce GABA distribution onto dopamine neurons, resulting in a spike in dopamine neuronal activity via a disengagement process. CB2 receptors, on the other hand, are mostly found in postsynaptic somatodendritic areas, and their activation reduces the rate of firing and the responsiveness of VTA and dopamine neurons in the brain [152]. CB2 receptors tend to be the key for underpinning protective effects on neurons, and addressing CB2 receptors could provide a unique therapeutic method for addressing neuropsychiatric and brain-related conditions, avoiding the normal CB1 receptor-mediated negative consequences [153,154] (Figure 3).

### 4.7. Cannabinoid Receptor 2 (CB2) in the Communication of Neuronal Signaling Pathways

As CB1 receptors are functioning, they reduce GABA discharge from presynaptic terminals, thereby reducing GABAergic neurons’ detrimental influence on the postsynaptic neurons. As a consequence of this disengagement process, postsynaptic neurons are activated. CB2 receptors, on the other hand, are mostly found in the cell nuclei of postsynaptic nerve cells [155,156]. As a result, the enrolment of such downstream CB2 receptors often result in a membrane potential hyperpolarization, which inhibits postsynaptic neuronal firing. Because of this discrepancy in distribution, CB1 and CB2 receptors activation produces opposite results. CB2 receptors expression reduces neuronal excitability via various methods. CB2 receptors expression reduces the excitability of neurons via a variety of methods. The CB2 receptors triggering, for example, operates for VTA dopamine neurons via modulating the K^+^ channel performance [155]. Intracellular CB2 receptors in frontal cortex neurons have connections to the Gq11-PLC-IP3 pathways, which initiates the development of Ca^2+^-dependent Cl^-^ channels.

As a consequence, the cell membrane becomes hyperpolarized, resulting in neural blocking [157]. Studies also evidence that CB2 receptor expression activates the Na^+^/Bicarbonate co-transporter in the hippocampus, specifically in the CA3/CA2 neuronal cells, resulting in a sustained synaptic hyperpolarization. Interestingly, CB2 receptors activation is autonomous, changing the input and output behavior of CA3 pyramidal neurons and modulating gamma oscillations in vivo [156]. The increased expression of CB2 receptors in midbrain dopamine neuronal cells suggests that they may have an impact on a variety of key behaviors connected to dopamine activity [158]. CB2 receptors have been linked to the regulation of hunger, managing weight [159,160,161,162], depression [163], stress [164], and schizophrenia-like behaviors [165]. Several studies have demonstrated how CB2 receptors in the brain have an important role in reducing drug dependence—mainly alcohol, nicotine, and psychotropic substances [166,167,168]. These results imply that CB2 receptors have a considerable influence on the mesocorticolimbic system and perform essential functions in a wide range of brain processes, including psychiatric, cognitive abilities, and neurological tasks.

### 4.8. Role of Cannabinoid Receptor 2 (CB2) in the Modulation of Neuronal Physiology

Previous data suggested that CB2 receptors have been found in immune cell populations trafficking in the circulation, the spleen, and macrophage-derived cells, including cartilage cells and hepatocellular Kupffer cells [146,169]. Unlike CB1 receptors, which are present throughout the CNS, CB2 receptors are generally located in the brainstem and hippocampus CA2/3 pyramidal cells under normal physiological circumstances [156,170]. Nevertheless, if there is an inflammatory condition or destruction, the level of expression of CB2 receptors in activated microglial cells inside the CNS can be dramatically elevated [141,156,171,172,173,174,175,176,177,178]. These cells, which comprise indigenous microglia, perivascular microglia, astrocytes, and oligodendrocytes, account for more than 70% of the total cell population in the brain and spinal cord. They serve as the initial defense against inflammation and other forms of damage [179,180]. Microglia are the brain’s innate immune system cells and play a crucial part in both health and disease [141].

Microglia play an important role in initiating and maintaining synaptic remodeling inside neuronal cells under normal physiological circumstances. This is accomplished by changes in the immediate environment and changes in synapse architecture [181,182,183,184,185]. In cases of neurological pain, activated microglia in the nerve roots express CB2 mRNA [181]. Particularly, CB2 receptor activity is increased in the dorsal horn in a variety of neurological pain scenarios, involving regional damage to neurons, chemotherapy-induced neurological pain, and persistent post-ischemia pain. This increased expression is associated with microglia that have been activated [175,181,186,187,188,189]. CB2 receptors have been identified in the autopsy brain tissue of Alzheimer’s patients [190,191]. Additionally, receptors for CB2 were found in high concentrations along with high specificity in microglial cells linked to neuritic plaques [171]. Furthermore, CB2 receptors are upregulated in activated microglia cells from mouse models of numerous diseases, including Alzheimer’s disease, Huntington’s condition, and multiple neurological disorders [192]. A negative feedback process helps to restore homeostasis in numerous physiological stress circumstances, like wound healing. The Toll-like receptor (TLR) stimulation inside microglial cells triggers a release of suppression systems’ cytokine signaling molecules [193], while members of the tumor necrosis factor-alpha (TNFα)-induced protein 8 families are known to control responses to inflammation [194].

Enhanced CB2 receptor manifestation and expression could indicate an aggressive approach to limit or modulate the inflammatory process. The analogy might be related to the way tumor suppressor genes limit cell proliferation and how ephrin receptors in the brain can have different actions depending on the conditions [195]. Several studies have shown that CB2 receptor stimulation helps to reduce short-term inflammation [196,197,198,199,200,201]. Whenever the CB2 receptor system is activated, it suppresses neuroinflammatory signaling pathways and restores glial activity to normal—transitioning from a pro-inflammatory to an anti-inflammatory condition. CB2 receptors additionally influence the triggering of ERK1/2 (extracellular signal-regulated protein kinases) [202]. Earlier studies also evidence that toll-like receptor 2 (TLR2) and TLR4 have been linked to ERK1/2 amplification [203]. Experimental evidence shows that anandamide, which is an endocannabinoid, works via the MAPK pathway inside the CNS immune system. This strategy attempts to reduce the extent of inflammation and limit immunological responses associated with neurotoxicity [202].

**Figure 4:**
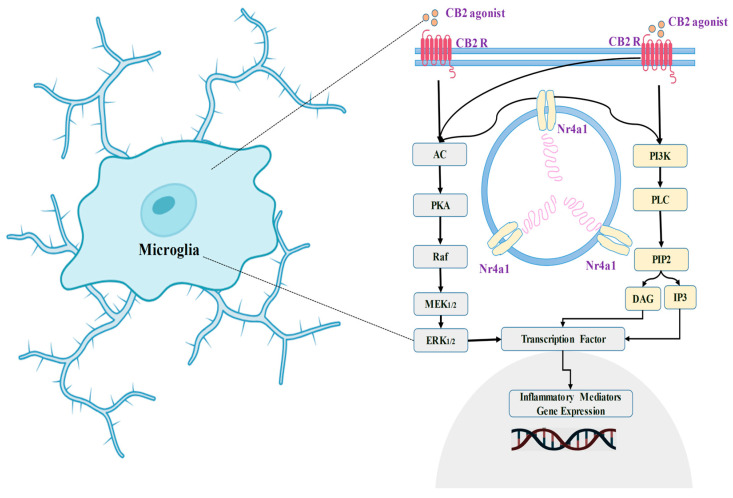
Location of CB2 receptor in the microglia, and its physiological retorts.

A prior investigation has shown that using a particular CB2 agonist named 1-(3-benzyl-3-methyl-2,3-dihydro-1-benzofuran-6-yl) carbonyl) piperidine (MDA7) [204] affects the expression of gene modulation in the neuroinflammatory state generated by paclitaxel. This is evidenced by the considerably reduced expression of TLR2, CB2 receptors, and ERK1/2 [205]. In addition, the use of MDA7 has been linked to changes in cerebral glutamatergic communication [205] (Figure 4).

### 4.9. The Involvement of the Cannabinoid Receptor 2 (CB2) in Synaptic Modulation

CB2 was first identified as a receptor found in distal macrophages [169]. Its existence in the brain was later validated by methods such as RT-PCR, in situ hybridization, and immunofluorescence studies. Regardless of the fact that CB2 levels throughout the brain are substantially less than those in the immune system [206], the receptor itself is found in microglial cells but not in astrocytes [207]. CB2 transcription is significantly increased in the face of prolonged pain [181,208,209]. CB2 was discovered within both astrocytes linked to neuritic deposits and microglia [171]. A new investigation identified that CB2 in the brainstem is biologically linked to vomiting control in collaboration with CB1 [210]. Previous research, nevertheless, demonstrated that CB2 mRNA was not found in the brain using methods such as RT-PCR and immunoblotting procedures [211].

A number of investigations have found neuronal CB2 activity in various brain locations [71,211,212], with CB2 being found in cell bodies of neurons and dendrites but not in synaptic endpoints [211]. Researchers effectively identified the existence of CB1 and CB2 mRNAs in the cerebral cortex of rats and mice using particular probes and primers. Researchers also provided evidence demonstrating the existence of CB2 receptors in numerous areas of the adult rat brain. The researchers used multiple antibodies from various sources, all engineered to target a distinct epitope on the CB2 receptor. When these antibodies were used to analyze the lymph nodes and other brain areas of rodents, they produced very comparable marking characteristics. The analogous antibody staining features seen in the lymph nodes and cerebellum provide evidence of this relationship. CB2-positive activity was also found in neurons optimistic for neuronal-specific enolase (NSE) in basic hippocampal preparations. The Western blot tests using extracts from the rodent’s spleen and brain revealed similar lines at the predicted molecular mass, which corresponded to the anticipated magnitude of the CB2 receptor. Immunohistochemical examinations revealed strong CB2 antibody staining across multiple neurons as well as glial cells throughout the brain.

### 4.10. Cannabinoid Receptor 2 (CB2) Gene Expression in the Brain

Earlier findings postulated that CB2 is located on chromosome 4 in rodents and on 1p36 in humans. Its genomic organization is straightforward, with a single translating exon. CB2 belongs to the GPCR receptor class and is made up of a single polypeptide chain with seven transmembrane α-helices. A glycosylated N-terminus is outside the cell, whereas an uninvolved C-terminus is inside. The CB2 region has only 44% nucleotide sequence analogy to the CB1 receptor. Whereas CB1 receptors are found in the brain, there is insufficient proof of CB2 binding to receptors, proteins, or mRNA in neurons [46]. Nonetheless, considerable quantitative evidence reveals that CB2 receptors are present in cultivated cerebral cell granules [213]. Advancement in cannabis pharmacotherapy has resulted in the discovery of several specialized antagonists and agonists that target these receptor categories [214]. The samples of proteins comprising rodent brain and spleen lysates revealed a conspicuous band at around 53 kDa, as well as further identifiable bands at roughly 37 kDa and 75 kDa. This discovery is consistent with current research [210]. It was additionally demonstrated that the location of CB2 receptors is particular by using three unique anti-CB2 antibodies, which were isolated according to their specificity, and targeting various amino acid endings, leading to equivalent marking trends.

The stains demonstrating the existence of CB2 receptors were ubiquitous throughout the cerebral cortex segments studied, including the orbital cortex, cerebral cortex, visual motor cortex, and auditory cortex. In layers III and V, the cell soma and top sections of pyramid-shaped neurons are heavily stained. In pyramid-shaped neurons in the hippocampus allocortex, an intermediate to significant CB2 immunostaining trend was seen. Such marking is prominent in the hippocampal CA2 and CA3 areas whereas it was less visible in the subiculum [208,210]. CB2 immunoreactivity was additionally detected in synapses situated in the stratum oriens and stratum radiatum. In addition, numerous glial cells showed immunostaining. Distinct neuronal soma-like and process-like CB2 staining patterns have been identified in the thalamus. The bulk of thalamic nuclei exhibited staining similar to cell bodies. Notably, well-defined processes were labeled within the reticular thalamic nucleus, featuring a dense network of fibers with CB2-like immunoreactivity. Nonetheless, CB2-positive labeling was seen in the cell nuclei of the ventral posterior thalamus region [215]. These structures are around 10–20 m in size.

The lateral posterior thalamic nucleus, the posterior thalamic nuclear category, including the paracentral thalamic nucleus all showed significant CB2 cell body marking. Cell bodies with fairly abundant CB2 immunopositivity were seen in the periaqueductal grey area. The greatest degree of staining was detected in the para-trochlear nucleus, the paralemniscal nucleus, and the red nuclei. The pontine nucleus constituted the most significant CB2-positive area inside the pons [216]. The pontine nucleus was defined by CB2-positive staining. The highly stained pathway seems to be an astrocyte or microglial process. CB2-like immunoreactivity was seen in substantia nigra pars reticulata, with clear labeling in the cell body, and some neuronal processes were noticeable. CB2-positive interneurons are larger than 20 m in size. The CB2-positive labeling appeared across the cerebellum lobes. Purkinje cell bodies were highly tarnished, but dendrites in the cellular tier were just faintly labeled. Numerous heavily stained fine puncta were seen in the molecular layer [206].

## 5. Discussion

Neuronal inflammation leads to swelling, damage, and neurological issues, including memory loss and neurodegenerative disorders. Conditions like ischemia, Alzheimer’s disease, Parkinson’s disease, mental health disorders, neurodevelopmental diseases, and immune-related ailments are influenced by this inflammation, involving microglia and mast cells, impacting neurological health and pain. Non-neuronal CNS cells play a crucial role in initiating, sustaining, and mitigating neuroinflammation, damage, and pain. Mast cells and microglia are key neuro-immune defenders in the brain, partnering with astrocytes to connect distant immune signals with the CNS during inflammation. Cannabis triggers diverse effects, like heightened senses, increased heart rate, pain relief, impaired focus, nausea reduction, and appetite increase. CB1 and CB2 GPCRs, distributed in the body, particularly CB1 in the brain, influence neurotransmitter release; CB2, recently found in CNS microglia and neurons, exhibits diverse concentrations, e.g., in the globus pallidus, substantia nigra, and hippocampus. Axonal CB1 receptors are linked to GABAergic nerve cells, specifically those in cholecystokinin-containing basket cells, impacting GABAergic synaptic terminals, particularly in hippocampal neurons. CB1 receptor activation in neural networks results in a reduced cAMP synthesis, an inhibited Ca^2+^ influx via N- and P/Q-type channels, and the activation of MAP kinase and possibly c-Jun N-terminal kinase. Cannabinoids also affect the release of slower neurotransmitters like opiates, acetylcholine, dopamine, and noradrenaline, with CB1 receptor immunoreactivity strongly connected to mRNA in GABAergic neurons. CB1 receptors inhibit hippocampal synaptic plasticity, including LTP and LTD, via a receptor-dependent mechanism. Studies using CB1 receptor-deficient mice suggest that endocannabinoids consistently hinder synaptic remodeling, possibly involving anandamide as an intrinsic messenger for cognitive regulation. CB1 receptor mRNA, assessed through RT-PCR, is present in striatal and extra-striatal regions like the globus pallidus and substantia nigra in the brain with Parkinson’s disease, with lowered levels in the neurons of the caudate nucleus and dorsal putamen. Research in neurological conditions reveals the unique presence of CB2 in microglia cells in disorders like Alzheimer’s, Multiple Sclerosis, and ALS. CB2 stimulation, akin to CB1, fosters adult synaptogenesis and potentially influences blood-brain barrier permeability. The evidence underscores the CB2 receptor’s involvement in mental and neurological conditions, with distinct brain distribution mainly in postsynaptic neuronal somatodendritic regions, leading to distinct effects compared to CB1 receptors. In contrast, CB2 receptors are predominantly located within the cell nuclei of postsynaptic neurons, leading to downstream activation that hyperpolarizes the membrane potential and inhibits neuronal firing. Neuronal excitability reduction is achieved through varied methods by CB2 receptor expression, such as the modulation of K^+^ channels for VTA dopamine neurons and involvement in Gq11-PLC-IP3 pathways in frontal cortex neurons. CB2 receptors also impact hunger, weight, depression, stress, and behaviors resembling schizophrenia through various mechanisms. Numerous studies indicate that CB2 receptors in the brain play a pivotal role in curbing addiction, particularly to alcohol, nicotine, and psychotropic substances. CB2 receptors are found abundantly in microglial cells linked to neuritic plaques in the brain tissue of Alzheimer’s patients. CB1 and CB2 mRNAs are present in the cerebral cortex of rodents and mice, as identified by specific probes and primers, with CB2 receptors located throughout various brain regions in adult rats. Structurally, CB2 is a GPCR receptor with a single polypeptide chain comprising seven transmembrane α-helices, originating from chromosome 4 in rodents and 1p36 in humans. 

## 6. Future Prospective

The future perspective of this review encompasses several avenues of exploration. Firstly, further investigations into the intricate relationship between neuroinflammation and various neurological conditions, such as neurodegenerative disorders, mental health issues, and immune-related ailments, could yield insights into potential therapeutic interventions. Understanding the roles of microglia, mast cells, and non-neuronal CNS cells in mitigating neuroinflammation and pain could lead to novel strategies for managing these conditions. Additionally, the modulation of CB1 and CB2 receptors in various brain regions appears to be a promising area for future research. Investigating how these receptors influence neurotransmitter release, synaptic plasticity, and neural excitability could uncover new pathways for addressing neurological disorders associated with neuroinflammation, neurodegeneration, and cognitive dysregulation.

## Data Availability

Data are contained within the article.

## References

[1] Abraham J., Johnson R.W. (2009). Consuming a Diet Supplemented with Resveratrol Reduced Infection-Related Neuroinflammation and Deficits in Working Memory in Aged Mice. Rejuvenation Res..

[2] Aungst S.L., Kabadi S.V., Thompson S.M., Stoica B.A., Faden A.I. (2014). Repeated Mild Traumatic Brain Injury Causes Chronic Neuroinflammation, Changes in Hippocampal Synaptic Plasticity, and Associated Cognitive Deficits. J. Cereb. Blood Flow Metab..

[3] Kielian T. (2016). Multifaceted Roles of Neuroinflammation: The Need to Consider Both Sides of the Coin. J. Neurochem..

[4] Kempuraj D., Thangavel R., Selvakumar G.P., Zaheer S., Ahmed M.E., Raikwar S.P., Zahoor H., Saeed D., Natteru P.A., Iyer S. (2017). Brain and Peripheral Atypical Inflammatory Mediators Potentiate Neuroinflammation and Neurodegeneration. Front. Cell. Neurosci..

[5] Freeman L.C., Ting J.P. (2016). The Pathogenic Role of the Inflammasome in Neurodegenerative Diseases. J. Neurochem..

[6] Radtke F.A., Chapman G., Hall J., Syed Y.A. (2017). Modulating Neuroinflammation to Treat Neuropsychiatric Disorders. Biomed Res. Int..

[7] Bjorklund G., Saad K., Chirumbolo S., Kern J., Geier D., Geier M., Urbina M. (2016). Immune Dysfunction and Neuroinflammation in Autism Spectrum Disorder. Acta Neurobiol. Exp..

[8] Terrone G., Salamone A., Vezzani A. (2017). Inflammation and Epilepsy: Preclinical Findings and Potential Clinical Translation. Curr. Pharm. Des..

[9] Vezzani A. (2013). Fetal Brain Inflammation May Prime Hyperexcitability and Behavioral Dysfunction Later in Life. Ann. Neurol..

[10] Calcia M.A., Bonsall D.R., Bloomfield P.S., Selvaraj S., Barichello T., Howes O.D. (2016). Stress and Neuroinflammation: A Systematic Review of the Effects of Stress on Microglia and the Implications for Mental Illness. Psychopharmacology.

[11] Schwartz M., Deczkowska A. (2016). Neurological Disease as a Failure of Brain–Immune Crosstalk: The Multiple Faces of Neuroinflammation. Trends Immunol..

[12] Skaper S.D. (2016). Mast Cell–Glia Dialogue in Chronic Pain and Neuropathic Pain: Blood-Brain Barrier Implications. CNS Neurol. Disord. Targets.

[13] Frasca D., Blomberg B.B., Paganelli R. (2017). Aging, Obesity, and Inflammatory Age-Related Diseases. Front. Immunol..

[14] Skaper S.D. (2015). Commentary: Low-Grade Non-Resolving Neuroinflammation: Age Does Matter. CNS Neurol. Disord. Targets.

[15] Basavarajappa B.S., Shivakumar M., Joshi V., Subbanna S. (2017). Endocannabinoid System in Neurodegenerative Disorders. J. Neurochem..

[16] Schurman L.D., Lichtman A.H. (2017). Endocannabinoids: A Promising Impact for Traumatic Brain Injury. Front. Pharmacol..

[17] Ranieri R., Laezza C., Bifulco M., Marasco D., Malfitano A.M. (2015). Endocannabinoid System in Neurological Disorders. Recent Patents CNS Drug Discov..

[18] Tsuboi K., Uyama T., Okamoto Y., Ueda N. (2018). Endocannabinoids and Related N-Acylethanolamines: Biological Activities and Metabolism. Inflamm. Regen..

[19] Gaoni Y., Mechoulam R. (1964). Isolation, Structure, and Partial Synthesis of an Active Constituent of Hashish. J. Am. Chem. Soc..

[20] Chaperon F., Thiebot M.-H. (1999). Behavioral Effects of Cannabinoid Agents in Animals. Crit. Rev. Neurobiol..

[21] Hollister L.E. (1986). Health Aspects of Cannabis. Pharmacol. Rev..

[22] Mechoulam R. (1999). Recent Advantages in Cannabinoid Research. Complement. Med. Res..

[23] Ameri A. (1999). The Effects of Cannabinoids on the Brain. Prog. Neurobiol..

[24] Pertwee R.G. (2001). Cannabinoid Receptors and Pain. Prog. Neurobiol..

[25] Harris L.S., Dewey W.L., Razdan R.K. (1977). Cannabis: Its Chemistry, Pharmacology, and Toxicology. Drug Addiction II: Amphetamine, Psychology, and Marihuana Dependence.

[26] Mechoulam R. (2019). Cannabinoids as Therapeutic Agents.

[27] Razdan R.K. (1986). Structure-Activity Relationships in Cannabinoids. Pharmacol. Rev..

[28] Dewey W.L. (1986). Cannabinoid Pharmacology. Pharmacol. Rev..

[29] Wilson R.S., May E.L. (1975). Analgesic Properties of the tetrahydrocannabinols, Their Metabolites, and Analogs. J. Med. Chem..

[30] Wilson R.S., May E.L., Martin B.R., Dewey W.L. (1976). 9-Nor-9-Hydroxyhexahydrocannabinols. Synthesis, Some Behavioral and Analgesic Properties, and Comparison with the Tetrahydrocannabinols. J. Med. Chem..

[31] Wilson R.S., May E.L., Dewey W.L. (1979). Some 9-Hydroxycannabinoid-like Compounds. Synthesis and Evaluation of Analgesic and Behavioral Properties. J. Med. Chem..

[32] Zuardi A.W. (2006). History of Cannabis as a Medicine: A Review. Brazilian J. Psychiatry.

[33] Mechoulam R. (1986). Cannabinoids as Therapeutic Agents.

[34] Mechoulam R., Shani A., Edery H., Grunfeld Y. (1970). Chemical Basis of Hashish Activity. Science.

[35] Russo E., Guy G.W. (2006). A Tale of Two Cannabinoids: The Therapeutic Rationale for Combining Tetrahydrocannabinol and Cannabidiol. Med. Hypotheses.

[36] O’Shaughnessy W.B. (1843). On the Preparations of the Indian Hemp, or Gunjah: Cannabis Indica Their Effects on the Animal System in Health, and Their Utility in the Treatment of Tetanus and Other Convulsive Diseases. Prov. Med. J. Retrosp. Med. Sci..

[37] Crippa J.A.S., Zuardi A.W., Hallak J.E.C. (2010). Therapeutical Use of the Cannabinoids in Psychiatry. Brazilian J. Psychiatry.

[38] Matias I., Di Marzo V. (2007). Endocannabinoids and the control of energy balance. Trends Endocrinol. Metab..

[39] Di Marzo V., De Petrocellis L. (2012). Why Do Cannabinoid Receptors Have More than One Endogenous Ligand?. Philos. Trans. R. Soc. B Biol. Sci..

[40] Magotti P., Bauer I., Igarashi M., Babagoli M., Marotta R., Piomelli D., Garau G. (2015). Structure of Human N-Acylphosphatidylethanolamine-Hydrolyzing Phospholipase D: Regulation of Fatty Acid Ethanolamide Biosynthesis by Bile Acids. Structure.

[41] Sugiura T., Kobayashi Y., Oka S., Waku K. (2002). Biosynthesis and Degradation of Anandamide and 2-Arachidonoylglycerol and Their Possible Physiological Significance. Prostaglandins, Leukot. Essent. Fat. Acids.

[42] Mackie K. (2008). Cannabinoid Receptors: Where They Are and What They Do. J. Neuroendocrinol..

[43] Akerman S., Holland P.R., Lasalandra M.P., Goadsby P.J. (2013). Endocannabinoids in the Brainstem Modulate Dural Trigeminovascular Nociceptive Traffic via CB1 and “Triptan” Receptors: Implications in Migraine. J. Neurosci..

[44] Jourdan T., Djaouti L., Demizieux L., Gresti J., Verges B., Degrace P. (2010). CB1 Antagonism Exerts Specific Molecular Effects on Visceral and Subcutaneous Fat and Reverses Liver Steatosis in Diet-Induced Obese Mice. Diabetes.

[45] Guo J., Ikeda S.R. (2004). Endocannabinoids Modulate N-Type Calcium Channels and G-Protein-Coupled Inwardly Rectifying Potassium Channels via CB1 Cannabinoid Receptors Heterologously Expressed in Mammalian Neurons. Mol. Pharmacol..

[46] Reggio P.H. (2010). Endocannabinoid Binding to the Cannabinoid Receptors: What Is Known and What Remains Unknown. Curr. Med. Chem..

[47] Osei-Hyiaman D., DePetrillo M., Pacher P., Liu J., Radaeva S., Bátkai S., Harvey-White J., Mackie K., Offertáler L., Wang L. (2005). Endocannabinoid Activation at Hepatic CB 1 Receptors Stimulates Fatty Acid Synthesis and Contributes to Diet-Induced Obesity. J. Clin. Investig..

[48] Miller A.M., Stella N. (2008). CB2 Receptor-mediated Migration of Immune Cells: It Can Go Either Way. Br. J. Pharmacol..

[49] Guindon J., Hohmann A.G. (2008). Cannabinoid CB2 Receptors: A Therapeutic Target for the Treatment of Inflammatory and Neuropathic Pain. Br. J. Pharmacol..

[50] Stella N. (2010). Cannabinoid and Cannabinoid-like Receptors in Microglia, Astrocytes, and Astrocytomas. Glia.

[51] Li Y., Kim J. (2017). Distinct Roles of Neuronal and Microglial CB2 Cannabinoid Receptors in the Mouse Hippocampus. Neuroscience.

[52] Chen D.-J., Gao M., Gao F.-F., Su Q.-X., Wu J. (2017). Brain Cannabinoid Receptor 2: Expression, Function and Modulation. Acta Pharmacol. Sin..

[53] Basu S., Dittel B.N. (2011). Unraveling the Complexities of Cannabinoid Receptor 2 (CB2) Immune Regulation in Health and Disease. Immunol. Res..

[54] Tambaro S., Casu M.A., Mastinu A., Lazzari P. (2014). Evaluation of Selective Cannabinoid CB1 and CB2 Receptor Agonists in a Mouse Model of Lipopolysaccharide-Induced Interstitial Cystitis. Eur. J. Pharmacol..

[55] Cristino L., De Petrocellis L., Pryce G., Baker D., Guglielmotti V., Di Marzo V. (2006). Immunohistochemical Localization of Cannabinoid Type 1 and Vanilloid Transient Receptor Potential Vanilloid Type 1 Receptors in the Mouse Brain. Neuroscience.

[56] Marzo V.D., Petrocellis L. (2010). De Endocannabinoids as Regulators of Transient Receptor Potential (TRP) Channels: A Further Opportunity to Develop New Endocannabinoid-Based Therapeutic Drugs. Curr. Med. Chem..

[57] De Petrocellis L., Di Marzo V. (2009). Role of Endocannabinoids and Endovanilloids in Ca^2+^ Signalling. Cell Calcium.

[58] Karaliota S., Siafaka-Kapadai A., Gontinou C., Psarra K., Mavri-Vavayanni M. (2009). Anandamide Increases the Differentiation of Rat Adipocytes and Causes PPARγ and CB1 Receptor Upregulation. Obesity.

[59] Berger J., Moller D.E. (2002). The Mechanisms of Action of PPARs. Annu. Rev. Med..

[60] Kota B.P., Huang T.H.-W., Roufogalis B.D. (2005). An Overview on Biological Mechanisms of PPARs. Pharmacol. Res..

[61] Huang J.V., Greyson C.R., Schwartz G.G. (2012). PPAR-γ as a Therapeutic Target in Cardiovascular Disease: Evidence and Uncertainty: Thematic Review Series: New Lipid and Lipoprotein Targets for the Treatment of Cardiometabolic Diseases. J. Lipid Res..

[62] Kelly R., Joers V., Tansey M.G., McKernan D.P., Dowd E. (2020). Microglial Phenotypes and Their Relationship to the Cannabinoid System: Therapeutic Implications for Parkinson’s Disease. Molecules.

[63] Morena M., Leitl K.D., Vecchiarelli H.A., Gray J.M., Campolongo P., Hill M.N. (2016). Emotional Arousal State Influences the Ability of Amygdalar Endocannabinoid Signaling to Modulate Anxiety. Neuropharmacology.

[64] Vecchiarelli H.A., Morena M., Keenan C.M., Chiang V., Tan K., Qiao M., Leitl K., Santori A., Pittman Q.J., Sharkey K.A. (2021). Comorbid Anxiety-like Behavior in a Rat Model of Colitis Is Mediated by an Upregulation of Corticolimbic Fatty Acid Amide Hydrolase. Neuropsychopharmacology.

[65] Šimončičová E., de Andrade E.G., Vecchiarelli H.A., Awogbindin I.O., Delage C.I., Tremblay M.-È. (2022). Present and Future of Microglial Pharmacology. Trends Pharmacol. Sci..

[66] St-Pierre M.-K., VanderZwaag J., Loewen S., Tremblay M.-È. (2022). All Roads Lead to Heterogeneity: The Complex Involvement of Astrocytes and Microglia in the Pathogenesis of Alzheimer’s Disease. Front. Cell. Neurosci..

[67] Pertwee R.G. (1997). Pharmacology of Cannabinoid CB1 and CB2 Receptors. Pharmacol. Ther..

[68] Shire D., Carillon C., Kaghad M., Calandra B., Rinaldi-Carmona M., Le Fur G., Caput D., Ferrara P. (1996). An Amino-Terminal Variant of the Central Cannabinoid Receptor Resulting from Alternative Splicing. J. Biol. Chem..

[69] Rinaldi-Carmona M., Calandra B., Shire D., Bouaboula M., Oustric D., Barth F., Casellas P., Ferrara P., Le Fur G. (1996). Characterization of Two Cloned Human CB1 Cannabinoid Receptor Isoforms. J. Pharmacol. Exp. Ther..

[70] Griffin G., Wray E.J., Tao Q., McAllister S.D., Rorrer W.K., Aung M., Martin B.R., Abood M.E. (1999). Evaluation of the Cannabinoid CB2 Receptor-Selective Antagonist, SR144528: Further Evidence for Cannabinoid CB2 Receptor Absence in the Rat Central Nervous System. Eur. J. Pharmacol..

[71] Skaper S.D., Buriani A., Dal Toso R., Petrelli L., Romanello S., Facci L., Leon A. (1996). The ALIAmide Palmitoylethanolamide and Cannabinoids, but Not Anandamide, Are Protective in a Delayed Postglutamate Paradigm of Excitotoxic Death in Cerebellar Granule Neurons. Proc. Natl. Acad. Sci. USA.

[72] Howlett A.C. (1995). Pharmacology of Cannabinoid Receptors. Annu. Rev. Pharmacol. Toxicol..

[73] Greenamyre J.T., Young A.B., Penney J.B. (1984). Quantitative Autoradiographic Distribution of L-[3H] Glutamate-Binding Sites in Rat Central Nervous System. J. Neurosci..

[74] Bowery N.G., Hudson A.L., Price G.W. (1987). GABAA and GABAB Receptor Site Distribution in the Rat Central Nervous System. Neuroscience.

[75] Herkenham M., Lynn A.B., Little M.D., Johnson M.R., Melvin L.S., de Costa B.R., Rice K.C. (1990). Cannabinoid receptor localization in brain. Proc. Natl. Acad. Sci. USA.

[76] Pettit D.A.D., Harrison M.P., Olson J.M., Spencer R.F., Cabral G.A. (1998). Immunohistochemical Localization of the Neural Cannabinoid Receptor in Rat Brain. J. Neurosci. Res..

[77] Tsou K., Brown S., Sañudo-Peña M.C., Mackie K., Walker J.M. (1998). Immunohistochemical Distribution of Cannabinoid CB1 Receptors in the Rat Central Nervous System. Neuroscience.

[78] Moldrich G., Wenger T. (2000). Localization of the CB1 Cannabinoid Receptor in the Rat Brain. An Immunohistochemical Study☆. Peptides.

[79] Egertová M., Elphick M.R. (2000). Localisation of Cannabinoid Receptors in the Rat Brain Using Antibodies to the Intracellular C-terminal Tail of CB1. J. Comp. Neurol..

[80] Tsou K., Mackie K., Sanudo-Pena M.C., Walker J.M. (1999). Cannabinoid CB1 Receptors Are Localized Primarily on Cholecystokinin-Containing GABAergic Interneurons in the Rat Hippocampal Formation. Neuroscience.

[81] Hájos N., Katona I., Naiem S.S., Mackie K., Ledent C., Mody I., Freund T.F. (2000). Cannabinoids Inhibit Hippocampal GABAergic Transmission and Network Oscillations. Eur. J. Neurosci..

[82] Katona I., Sperlagh B., Maglóczky Z., Santha E., Köfalvi A., Czirjak S., Mackie K., Vizi E.S., Freund T.F. (2000). GABAergic Interneurons Are the Targets of Cannabinoid Actions in the Human Hippocampus. Neuroscience.

[83] Katona I., Rancz E.A., Acsády L., Ledent C., Mackie K., Hájos N., Freund T.F. (2001). Distribution of CB1 Cannabinoid Receptors in the Amygdala and Their Role in the Control of GABAergic Transmission. J. Neurosci..

[84] Marsicano G., Lutz B. (1999). Expression of the Cannabinoid Receptor CB1 in Distinct Neuronal Subpopulations in the Adult Mouse Forebrain. Eur. J. Neurosci..

[85] Irving A.J., Coutts A.A., Harvey J., Rae M.G., Mackie K., Bewick G.S., Pertwee R.G. (2000). Functional Expression of Cell Surface Cannabinoid CB1 Receptors on Presynaptic Inhibitory Terminals in Cultured Rat Hippocampal Neurons. Neuroscience.

[86] Childers S.R., Deadwyler S.A. (1996). Role of Cyclic AMP in the Actions of Cannabinoid Receptors. Biochem. Pharmacol..

[87] Twitchell W., Brown S., Mackie K. (1997). Cannabinoids Inhibit N- and P/Q-Type Calcium Channels in Cultured Rat Hippocampal Neurons. J. Neurophysiol..

[88] Mu J., Zhuang S., Kirby M.T., Hampson R.E., Deadwyler S.A. (1999). Cannabinoid Receptors Differentially Modulate Potassium A and D Currents in Hippocampal Neurons in Culture. J. Pharmacol. Exp. Ther..

[89] Schweitzer P. (2000). Cannabinoids Decrease the K+ M-Current in Hippocampal CA1 Neurons. J. Neurosci..

[90] Glass M., Felder C.C. (1997). Concurrent Stimulation of Cannabinoid CB1 and Dopamine D2 Receptors Augments CAMP Accumulation in Striatal Neurons: Evidence for a Gs Linkage to the CB1 Receptor. J. Neurosci..

[91] Bouaboula M., Poinot-Chazel C., Bourrie B., Canat X., Calandra B., Rinaldi-Carmona M., Le Fur G., Casellas P. (1995). Activation of Mitogen-Activated Protein Kinases by Stimulation of the Central Cannabinoid Receptor CB1. Biochem. J..

[92] Rueda D., Galve-Roperh I., Haro A., Guzmán M. (2000). The CB1 Cannabinoid Receptor Is Coupled to the Activation of C-Jun N-Terminal Kinase. Mol. Pharmacol..

[93] Netzeband J.G., Conroy S.M., Parsons K.L., Gruol D.L. (1999). Cannabinoids Enhance NMDA-Elicited Ca^2+^ Signals in Cerebellar Granule Neurons in Culture. J. Neurosci..

[94] Rinaldi-Carmona M., Le Duigou A., Oustric D., Barth F., Bouaboula M., Carayon P., Casellas P., Le Fur G. (1998). Modulation of CB1 Cannabinoid Receptor Functions after a Long-Term Exposure to Agonist or Inverse Agonist in the Chinese Hamster Ovary Cell Expression System. J. Pharmacol. Exp. Ther..

[95] Coutts A.A., Anavi-Goffer S., Ross R.A., MacEwan D.J., Mackie K., Pertwee R.G., Irving A.J. (2001). Agonist-Induced Internalization and Trafficking of Cannabinoid CB1 Receptors in Hippocampal Neurons. J. Neurosci..

[96] Jin W., Brown S., Roche J.P., Hsieh C., Celver J.P., Kovoor A., Chavkin C., Mackie K. (1999). Distinct Domains of the CB1 Cannabinoid Receptor Mediate Desensitization and Internalization. J. Neurosci..

[97] Hsieh C., Brown S., Derleth C., Mackie K. (1999). Internalization and Recycling of the CB1 Cannabinoid Receptor. J. Neurochem..

[98] Hohmann A.G., Herkenham M. (1999). Cannabinoid Receptors Undergo Axonal Flow in Sensory Nerves. Neuroscience.

[99] Houser S.J., Eads M., Embrey J.P., Welch S.P. (2000). Dynorphin B and Spinal Analgesia: Induction of Antinociception by the Cannabinoids CP55, 940, Δ9-THC and Anandamide. Brain Res..

[100] Beinfeld M.C., Connolly K. (2001). Activation of CB1 Cannabinoid Receptors in Rat Hippocampal Slices Inhibits Potassium-Evoked Cholecystokinin Release, a Possible Mechanism Contributing to the Spatial Memory Defects Produced by Cannabinoids. Neurosci. Lett..

[101] Schlicker E., Timm J., Zentner J., Göthert M. (1997). Cannabinoid CB1 Receptor-Mediated Inhibition of Noradrenaline Release in the Human and Guinea-Pig Hippocampus. Naunyn-Schmiedeberg’s Arch. Pharmacol..

[102] Cadogan A., Alexander S.P.H., Boyd E.A., Kendall D.A. (1997). Influence of Cannabinoids on Electrically Evoked Dopamine Release and Cyclic AMP Generation in the Rat Striatum. J. Neurochem..

[103] Gifford A.N., Ashby C.R. (1996). Electrically Evoked Acetylcholine Release from Hippocampal Slices Is Inhibited by the Cannabinoid Receptor Agonist, WIN 55212-2, and Is Potentiated by the Cannabinoid Antagonist, SR 141716A. J. Pharmacol. Exp. Ther..

[104] Valverde O., Noble F., Beslot F., Daugé V., Fournié-Zaluski M., Roques B.P. (2001). Δ9-tetrahydrocannabinol Releases and Facilitates the Effects of Endogenous Enkephalins: Reduction in Morphine Withdrawal Syndrome without Change in Rewarding Effect. Eur. J. Neurosci..

[105] Coull M.A., Johnston A.T., Pertwee R.G., Davies S.N. (1997). Action of δ-9-Tetrahydrocannabinol on Gabaa Receptor-Mediated Responses in a Grease-Gap Recording Preparation of the Rat Hippocampal Slice. Neuropharmacology.

[106] Maneuf Y.P., Nash J.E., Crossman A.R., Brotchie J.M. (1996). Activation of the Cannabinoid Receptor by Δ9-Tetrahydrocannabinol Reduces γ-Aminobutyric Acid Uptake in the Globus Pallidus. Eur. J. Pharmacol..

[107] Herkenham M., Lynn A.B., Johnson M.R., Melvin L.S., de Costa B.R., Rice K.C. (1991). Characterization and Localization of Cannabinoid Receptors in Rat Brain: A Quantitative in Vitro Autoradiographic Study. J. Neurosci..

[108] Hoffman A.F., Lupica C.R. (2000). Mechanisms of Cannabinoid Inhibition of GABAASynaptic Transmission in the Hippocampus. J. Neurosci..

[109] Wilson R.I., Kunos G., Nicoll R.A. (2001). Presynaptic Specificity of Endocannabinoid Signaling in the Hippocampus. Neuron.

[110] Miller A.S., Walker J.M. (1995). Effects of a Cannabinoid on Spontaneous and Evoked Neuronal Activity in the Substantia Nigra Pars Reticulata. Eur. J. Pharmacol..

[111] Szabo B., Dörner L., Pfreundtner C., Nörenberg W., Starke K. (1998). Inhibition of GABAergic Inhibitory Postsynaptic Currents by Cannabinoids in Rat Corpus Striatum. Neuroscience.

[112] Chan P.K.Y., Chan S.C.Y., Yung W. (1998). Presynaptic Inhibition of GABAergic Inputs to Rat Substantia Nigra Pars Reticulata Neurones by a Cannabinoid Agonist. Neuroreport.

[113] Hoffman A.F., Lupica C.R. (2001). Direct Actions of Cannabinoids on Synaptic Transmission in the Nucleus Accumbens: A Comparison with Opioids. J. Neurophysiol..

[114] Manzoni O.J., Bockaert J. (2001). Cannabinoids Inhibit GABAergic Synaptic Transmission in Mice Nucleus Accumbens. Eur. J. Pharmacol..

[115] Vaughan C.W., McGregor I.S., Christie M.J. (1999). Cannabinoid Receptor Activation Inhibits GABAergic Neurotransmission in Rostral Ventromedial Medulla Neurons in Vitro. Br. J. Pharmacol..

[116] Vaughan C.W., Connor M., Bagley E.E., Christie M.J. (2000). Actions of Cannabinoids on Membrane Properties and Synaptic Transmission in Rat Periaqueductal Gray Neurons in Vitro. Mol. Pharmacol..

[117] Kreitzer A.C., Regehr W.G. (2001). Cerebellar Depolarization-Induced Suppression of Inhibition Is Mediated by Endogenous Cannabinoids. J. Neurosci..

[118] Takahashi K.A., Linden D.J. (2000). Cannabinoid Receptor Modulation of Synapses Received by Cerebellar Purkinje Cells. J. Neurophysiol..

[119] Caldwell D., Coutts A.A., Mackie K., Irving A.J. (1999). Cell Surface CB1 Receptors Are Expressed at Synaptic Terminals in Cultured Rat Cerebellar Granule Cells. J. Physiol.(London).

[120] Kreitzer A.C., Regehr W.G. (2001). Retrograde Inhibition of Presynaptic Calcium Influx by Endogenous Cannabinoids at Excitatory Synapses onto Purkinje Cells. Neuron.

[121] Maejima T., Ohno-Shosaku T., Kano M. (2001). Endogenous Cannabinoid as a Retrograde Messenger from Depolarized Postsynaptic Neurons to Presynaptic Terminals. Neurosci. Res..

[122] Lévénès C., Daniel H., Soubrié P., Crépel F. (1998). Cannabinoids Decrease Excitatory Synaptic Transmission and Impair Long-Term Depression in Rat Cerebellar Purkinje Cells. J. Physiol..

[123] Huang C., Lo S., Hsu K. (2001). Presynaptic Mechanisms Underlying Cannabinoid Inhibition of Excitatory Synaptic Transmission in Rat Striatal Neurons. J. Physiol..

[124] Robbe D., Alonso G., Duchamp F., Bockaert J., Manzoni O.J. (2001). Localization and Mechanisms of Action of Cannabinoid Receptors at the Glutamatergic Synapses of the Mouse Nucleus Accumbens. J. Neurosci..

[125] Shen M., Piser T.M., Seybold V.S., Thayer S.A. (1996). Cannabinoid Receptor Agonists Inhibit Glutamatergic Synaptic Transmission in Rat Hippocampal Cultures. J. Neurosci..

[126] Misner D.L., Sullivan J.M. (1999). Mechanism of Cannabinoid Effects on Long-Term Potentiation and Depression in Hippocampal CA1 Neurons. J. Neurosci..

[127] Paton G.S., Pertwee R.G., Davies S.N. (1998). Correlation between Cannabinoid Mediated Effects on Paired Pulse Depression and Induction of Long Term Potentiation in the Rat Hippocampal Slice. Neuropharmacology.

[128] Collins D.R., Pertwee R.G., Davies S.N. (1994). The Action of Synthetic Cannabinoids on the Induction of Long-Term Potentiation in the Rat Hippocampal Slice. Eur. J. Pharmacol..

[129] Terranova J.P., Michaud J.C., Fur G.L., Soubrié P. (1995). Inhibition of Long-Term Potentiation in Rat Hippocampal Slices by Anandamide and WIN55212-2: Reversal by SR141716 A, a Selective Antagonist of CB1 Cannabinoid Receptors. Naunyn Schmiedebergs Arch Pharmacol..

[130] Auclair N., Otani S., Soubrie P., Crepel F. (2000). Cannabinoids Modulate Synaptic Strength and Plasticity at Glutamatergic Synapses of Rat Prefrontal Cortex Pyramidal Neurons. J. Neurophysiol..

[131] Gerdeman G., Lovinger D.M. (2001). CB1 Cannabinoid Receptor Inhibits Synaptic Release of Glutamate in Rat Dorsolateral Striatum. J. Neurophysiol..

[132] Lazenka M.F., Selley D.E., Sim-Selley L.J. (2013). Brain Regional Differences in CB1 Receptor Adaptation and Regulation of Transcription. Life Sci..

[133] Sim-Selley L.J. (2003). Regulation of Cannabinoid CB1 Receptors in the Central Nervous System by Chronic Cannabinoids. Crit. Rev. Neurobiol..

[134] Villares J. (2007). Chronic Use of Marijuana Decreases Cannabinoid Receptor Binding and MRNA Expression in the Human Brain. Neuroscience.

[135] Hirvonen J., Goodwin R.S., Li C.-T., Terry G.E., Zoghbi S.S., Morse C., Pike V.W., Volkow N.D., Huestis M.A., Innis R. (2012). Reversible and Regionally Selective Downregulation of Brain Cannabinoid CB1 Receptors in Chronic Daily Cannabis Smokers. Mol. Psychiatry.

[136] Herdegen T., Leah J.D. (1998). Inducible and Constitutive Transcription Factors in the Mammalian Nervous System: Control of Gene Expression by Jun, Fos and Krox, and CREB/ATF Proteins. Brain Res. Rev..

[137] Howlett A.C., Barth F., Bonner T.I., Cabral G., Casellas P., Devane W.A., Felder C.C., Herkenham M., Mackie K., Martin B.R. (2002). International Union of Pharmacology. XXVII. Classification of Cannabinoid Receptors. Pharmacol. Rev..

[138] Kasukawa T., Masumoto K., Nikaido I., Nagano M., Uno K.D., Tsujino K., Hanashima C., Shigeyoshi Y., Ueda H.R. (2011). Quantitative Expression Profile of Distinct Functional Regions in the Adult Mouse Brain. PLoS ONE.

[139] Aymerich M.S., Aso E., Abellanas M.A., Tolon R.M., Ramos J.A., Ferrer I., Romero J., Fernández-Ruiz J. (2018). Cannabinoid Pharmacology/Therapeutics in Chronic Degenerative Disorders Affecting the Central Nervous System. Biochem. Pharmacol..

[140] Cassano T., Calcagnini S., Pace L., De Marco F., Romano A., Gaetani S. (2017). Cannabinoid Receptor 2 Signaling in Neurodegenerative Disorders: From Pathogenesis to a Promising Therapeutic Target. Front. Neurosci..

[141] Chung Y.C., Shin W.-H., Baek J.Y., Cho E.J., Baik H.H., Kim S.R., Won S.-Y., Jin B.K. (2016). CB2 Receptor Activation Prevents Glial-Derived Neurotoxic Mediator Production, BBB Leakage and Peripheral Immune Cell Infiltration and Rescues Dopamine Neurons in the MPTP Model of Parkinson’s Disease. Exp. Mol. Med..

[142] Palazuelos J., Ortega Z., Díaz-Alonso J., Guzmán M., Galve-Roperh I. (2012). CB2 Cannabinoid Receptors Promote Neural Progenitor Cell Proliferation via MTORC1 Signaling. J. Biol. Chem..

[143] Burdyga G., Lal S., Varro A., Dimaline R., Thompson D.G., Dockray G.J. (2004). Expression of Cannabinoid CB1 Receptors by Vagal Afferent Neurons Is Inhibited by Cholecystokinin. J. Neurosci..

[144] McCoy K.L., Matveyeva M., Carlisle S.J., Cabral G.A. (1999). Cannabinoid Inhibition of the Processing of Intact Lysozyme by Macrophages: Evidence for CB2 Receptor Participation. J. Pharmacol. Exp. Ther..

[145] Schatz A.R., Lee M., Condie R.B., Pulaski J.T., Kaminski N.E. (1997). Cannabinoid Receptors CB1 and CB2: A Characterization of Expression and Adenylate Cyclase Modulation within the Immune System. Toxicol. Appl. Pharmacol..

[146] Galiègue S., Mary S., Marchand J., Dussossoy D., Carrière D., Carayon P., Bouaboula M., Shire D., LE Fur G., Casellas P. (1995). Expression of Central and Peripheral Cannabinoid Receptors in Human Immune Tissues and Leukocyte Subpopulations. Eur. J. Biochem..

[147] Buckley N.E., McCoy K.L., Mezey É., Bonner T., Zimmer A., Felder C.C., Glass M., Zimmer A. (2000). Immunomodulation by Cannabinoids Is Absent in Mice Deficient for the Cannabinoid CB2 Receptor. Eur. J. Pharmacol..

[148] Buckley N.E. (2008). The Peripheral Cannabinoid Receptor Knockout Mice: An Update. Br. J. Pharmacol..

[149] Miller L.K., Devi L.A. (2011). The Highs and Lows of Cannabinoid Receptor Expression in Disease: Mechanisms and Their Therapeutic Implications. Pharmacol. Rev..

[150] Onaivi E.S., Ishiguro H., Gong J., Patel S., Meozzi P.A., Myers L., Perchuk A., Mora Z., Tagliaferro P.A., Gardner E. (2008). Functional Expression of Brain Neuronal CB2 Cannabinoid Receptors Are Involved in the Effects of Drugs of Abuse and in Depression. Ann. N. Y. Acad. Sci..

[151] Onaivi E.S., Ishiguro H., Gu S., Liu Q.-R. (2012). CNS Effects of CB2 Cannabinoid Receptors: Beyond Neuro-Immuno-Cannabinoid Activity. J. Psychopharmacol..

[152] Ma Z., Gao F., Larsen B., Gao M., Luo Z., Chen D., Ma X., Qiu S., Zhou Y., Xie J. (2019). Mechanisms of Cannabinoid CB(2) Receptor-Mediated Reduction of Dopamine Neuronal Excitability in Mouse Ventral Tegmental Area. EBioMedicine.

[153] Rathod S.S., Agrawal Y.O. Phytocannabinoids as Potential Multitargeting Neuroprotectants in Alzheimer’s Disease. Curr. Drug Res. Rev..

[154] Xin Q., Xu F., Taylor D.H., Zhao J.-F., Wu J. (2020). The Impact of Cannabinoid Type 2 Receptors (CB2Rs) in Neuroprotection against Neurological Disorders. Acta Pharmacol. Sin..

[155] Zhang H.-Y., Gao M., Liu Q.-R., Bi G.-H., Li X., Yang H.-J., Gardner E.L., Wu J., Xi Z.-X. (2014). Cannabinoid CB2 Receptors Modulate Midbrain Dopamine Neuronal Activity and Dopamine-Related Behavior in Mice. Proc. Natl. Acad. Sci. USA.

[156] Stempel A.V., Stumpf A., Zhang H.-Y., Özdoğan T., Pannasch U., Theis A.-K., Otte D.-M., Wojtalla A., Rácz I., Ponomarenko A. (2016). Cannabinoid Type 2 Receptors Mediate a Cell Type-Specific Plasticity in the Hippocampus. Neuron.

[157] den Boon F.S., Chameau P., Schaafsma-Zhao Q., van Aken W., Bari M., Oddi S., Kruse C.G., Maccarrone M., Wadman W.J., Werkman T.R. (2012). Excitability of Prefrontal Cortical Pyramidal Neurons Is Modulated by Activation of Intracellular Type-2 Cannabinoid Receptors. Proc. Natl. Acad. Sci. USA..

[158] Vlachou S., Panagis G. (2014). Regulation of Brain Reward by the Endocannabinoid System: A Critical Review of Behavioral Studies in Animals. Curr. Pharm. Des..

[159] Agudo J., Martin M., Roca C., Molas M., Bura A.S., Zimmer A., Bosch F., Maldonado R. (2010). Deficiency of CB2 Cannabinoid Receptor in Mice Improves Insulin Sensitivity but Increases Food Intake and Obesity with Age. Diabetologia.

[160] Ignatowska-Jankowska B., Jankowski M.M., Swiergiel A.H. (2011). Cannabidiol Decreases Body Weight Gain in Rats: Involvement of CB2 Receptors. Neurosci. Lett..

[161] Flake N.M., Zweifel L.S. (2012). Behavioral Effects of Pulp Exposure in Mice Lacking Cannabinoid Receptor 2. J. Endod..

[162] Emadi L., Jonaidi H., Hosseini Amir Abad E. (2011). The Role of Central CB2 Cannabinoid Receptors on Food Intake in Neonatal Chicks. J. Comp. Physiol. A.

[163] García-Gutiérrez M.S., Pérez-Ortiz J.M., Gutiérrez-Adán A., Manzanares J. (2010). Depression-resistant Endophenotype in Mice Overexpressing Cannabinoid CB2 Receptors. Br. J. Pharmacol..

[164] García-Gutiérrez M.S., Manzanares J. (2011). Overexpression of CB2 Cannabinoid Receptors Decreased Vulnerability to Anxiety and Impaired Anxiolytic Action of Alprazolam in Mice. J. Psychopharmacol..

[165] Ortega-Alvaro A., Aracil-Fernández A., García-Gutiérrez M.S., Navarrete F., Manzanares J. (2011). Deletion of CB2 Cannabinoid Receptor Induces Schizophrenia-Related Behaviors in Mice. Neuropsychopharmacology.

[166] Xi Z.-X., Peng X.-Q., Li X., Song R., Zhang H.-Y., Liu Q.-R., Yang H.-J., Bi G.-H., Li J., Gardner E.L. (2011). Brain Cannabinoid CB2 Receptors Modulate Cocaine’s Actions in Mice. Nat. Neurosci..

[167] Ortega-Álvaro A., Ternianov A., Aracil-Fernández A., Navarrete F., García-Gutiérrez M.S., Manzanares J. (2015). Role of Cannabinoid CB2 Receptor in the Reinforcing Actions of Ethanol. Addict. Biol..

[168] Navarrete F., Rodríguez-Arias M., Martín-García E., Navarro D., García-Gutiérrez M.S., Aguilar M.A., Aracil-Fernández A., Berbel P., Miñarro J., Maldonado R. (2013). Role of CB2 Cannabinoid Receptors in the Rewarding, Reinforcing, and Physical Effects of Nicotine. Neuropsychopharmacology.

[169] Munro S., Thomas K.L., Abu-Shaar M. (1993). Molecular Characterization of a Peripheral Receptor for Cannabinoids. Nature.

[170] Ofek O., Karsak M., Leclerc N., Fogel M., Frenkel B., Wright K., Tam J., Attar-Namdar M., Kram V., Shohami E. (2006). Peripheral Cannabinoid Receptor, CB2, Regulates Bone Mass. Proc. Natl. Acad. Sci. USA.

[171] Benito C., Núñez E., Tolón R.M., Carrier E.J., Rábano A., Hillard C.J., Romero J. (2003). Cannabinoid CB2 Receptors and Fatty Acid Amide Hydrolase Are Selectively Overexpressed in Neuritic Plaque-Associated Glia in Alzheimer’s Disease Brains. J. Neurosci..

[172] Benito C., Kim W.-K., Chavarría I., Hillard C.J., Mackie K., Tolón R.M., Williams K., Romero J. (2005). A Glial Endogenous Cannabinoid System Is Upregulated in the Brains of Macaques with Simian Immunodeficiency Virus-Induced Encephalitis. J. Neurosci..

[173] Ramírez B.G., Blázquez C., Del Pulgar T.G., Guzmán M., de Ceballos M.L. (2005). Prevention of Alzheimer’s Disease Pathology by Cannabinoids: Neuroprotection Mediated by Blockade of Microglial Activation. J. Neurosci..

[174] Ashton J.C., Rahman R.M.A., Nair S.M., Sutherland B.A., Glass M., Appleton I. (2007). Cerebral Hypoxia-Ischemia and Middle Cerebral Artery Occlusion Induce Expression of the Cannabinoid CB2 Receptor in the Brain. Neurosci. Lett..

[175] Naguib M., Xu J.J., Diaz P., Brown D.L., Cogdell D., Bie B., Hu J., Craig S., Hittelman W.N. (2012). Prevention of Paclitaxel-Induced Neuropathy through Activation of the Central Cannabinoid Type 2 Receptor System. Anesth. Analg..

[176] Wu J., Bie B., Yang H., Xu J.J., Brown D.L., Naguib M. (2013). Activation of the CB2 Receptor System Reverses Amyloid-Induced Memory Deficiency. Neurobiol. Aging.

[177] Wu J., Hocevar M., Foss J.F., Bie B., Naguib M. (2017). Activation of CB2 Receptor System Restores Cognitive Capacity and Hippocampal Sox2 Expression in a Transgenic Mouse Model of Alzheimer’s Disease. Eur. J. Pharmacol..

[178] Xu J., Tang Y., Xie M., Bie B., Wu J., Yang H., Foss J.F., Yang B., Rosenquist R.W., Naguib M. (2016). Activation of Cannabinoid Receptor 2 Attenuates Mechanical Allodynia and Neuroinflammatory Responses in a Chronic Post-ischemic Pain Model of Complex Regional Pain Syndrome Type I in Rats. Eur. J. Neurosci..

[179] Romero-Sandoval E.A., Horvath R.J., DeLeo J.A. (2008). Neuroimmune Interactions and Pain: Focus on Glial-Modulating Targets. Curr. Opin. Investig. drugs.

[180] Villacampa N., Heneka M.T. (2018). Microglia: You’ll Never Walk Alone!. Immunity.

[181] Zhang J., Hoffert C., Vu H.K., Groblewski T., Ahmad S., O’Donnell D. (2003). Induction of CB2 Receptor Expression in the Rat Spinal Cord of Neuropathic but Not Inflammatory Chronic Pain Models. Eur. J. Neurosci..

[182] Schafer D.P., Stevens B. (2013). Phagocytic Glial Cells: Sculpting Synaptic Circuits in the Developing Nervous System. Curr. Opin. Neurobiol..

[183] Schafer D.P., Lehrman E.K., Kautzman A.G., Koyama R., Mardinly A.R., Yamasaki R., Ransohoff R.M., Greenberg M.E., Barres B.A., Stevens B. (2012). Microglia Sculpt Postnatal Neural Circuits in an Activity and Complement-Dependent Manner. Neuron.

[184] Tremblay M.-È., Majewska A.K. (2011). A Role for Microglia in Synaptic Plasticity?. Commun. Integr. Biol..

[185] Tremblay M.-È., Lowery R.L., Majewska A.K. (2010). Microglial Interactions with Synapses Are Modulated by Visual Experience. PLoS Biol..

[186] Zhang W., Lu L., Lai Q., Zhu B., Li Z., Xu Y., Shao Z., Herrup K., Moore B.S., Ross A.C. (2016). Family-Wide Structural Characterization and Genomic Comparisons Decode the Diversity-Oriented Biosynthesis of Thalassospiramides by Marine Proteobacteria. J. Biol. Chem..

[187] Wotherspoon G., Fox A., McIntyre P., Colley S., Bevan S., Winter J. (2005). Peripheral Nerve Injury Induces Cannabinoid Receptor 2 Protein Expression in Rat Sensory Neurons. Neuroscience.

[188] Romero-Sandoval A., Nutile-McMenemy N., DeLeo J.A. (2008). Spinal Microglial and Perivascular Cell Cannabinoid Receptor Type 2 Activation Reduces Behavioral Hypersensitivity without Tolerance after Peripheral Nerve Injury. J. Am. Soc. Anesthesiol..

[189] Svíženská I.H., Brázda V., Klusáková I., Dubový P. (2013). Bilateral Changes of Cannabinoid Receptor Type 2 Protein and MRNA in the Dorsal Root Ganglia of a Rat Neuropathic Pain Model. J. Histochem. Cytochem..

[190] Solas M., Francis P.T., Franco R., Ramirez M.J. (2013). CB2 Receptor and Amyloid Pathology in Frontal Cortex of Alzheimer’s Disease Patients. Neurobiol. Aging.

[191] Tolón R.M., Núñez E., Pazos M.R., Benito C., Castillo A.I., Martínez-Orgado J.A., Romero J. (2009). The Activation of Cannabinoid CB2 Receptors Stimulates in Situ and in Vitro Beta-Amyloid Removal by Human Macrophages. Brain Res..

[192] Zarruk J.G., Fernández-López D., García-Yébenes I., García-Gutiérrez M.S., Vivancos J., Nombela F., Torres M., Burguete M.C., Manzanares J., Lizasoain I. (2012). Cannabinoid Type 2 Receptor Activation Downregulates Stroke-Induced Classic and Alternative Brain Macrophage/Microglial Activation Concomitant to Neuroprotection. Stroke.

[193] Sun H., Gong S., Carmody R.J., Hilliard A., Li L., Sun J., Kong L., Xu L., Hilliard B., Hu S. (2008). TIPE2, a Negative Regulator of Innate and Adaptive Immunity That Maintains Immune Homeostasis. Cell.

[194] Ertl N.G., O’Connor W.A., Papanicolaou A., Wiegand A.N., Elizur A. (2016). Transcriptome Analysis of the Sydney Rock Oyster, Saccostrea Glomerata: Insights into Molluscan Immunity. PLoS ONE.

[195] Hou S.T., Jiang S.X., Smith R.A. (2008). Permissive and Repulsive Cues and Signalling Pathways of Axonal Outgrowth and Regeneration. Int. Rev. Cell Mol. Biol..

[196] Fernandez-Ruiz J., Romero J., Velasco G., Tolon R.M., Ramos J.A., Guzman M. (2007). Cannabinoid CB2 Receptor: A New Target for Controlling Neural Cell Survival?. Trends Pharmacol. Sci..

[197] Lotersztajn S., Teixeira-Clerc F., Julien B., Deveaux V., Ichigotani Y., Manin S., Tran-Van-Nhieu J., Karsak M., Zimmer A., Mallat A. (2008). CB2 Receptors as New Therapeutic Targets for Liver Diseases. Br. J. Pharmacol..

[198] Wright K.L., Duncan M., Sharkey K.A. (2008). Cannabinoid CB2 Receptors in the Gastrointestinal Tract: A Regulatory System in States of Inflammation. Br. J. Pharmacol..

[199] Merighi S., Gessi S., Varani K., Fazzi D., Mirandola P., Borea P.A. (2012). Cannabinoid CB2 Receptor Attenuates Morphine-induced Inflammatory Responses in Activated Microglial Cells. Br. J. Pharmacol..

[200] Arévalo-Martín A., García-Ovejero D., Gomez O., Rubio-Araiz A., Navarro-Galve B., Guaza C., Molina-Holgado E., Molina-Holgado F. (2008). CB2 Cannabinoid Receptors as an Emerging Target for Demyelinating Diseases: From Neuroimmune Interactions to Cell Replacement Strategies. Br. J. Pharmacol..

[201] Romero-Sandoval A., Eisenach J.C. (2007). Spinal Cannabinoid Receptor Type 2 Activation Reduces Hypersensitivity and Spinal Cord Glial Activation after Paw Incision. J. Am. Soc. Anesthesiol..

[202] Eljaschewitsch E., Witting A., Mawrin C., Lee T., Schmidt P.M., Wolf S., Hoertnagl H., Raine C.S., Schneider-Stock R., Nitsch R. (2006). The Endocannabinoid Anandamide Protects Neurons during CNS Inflammation by Induction of MKP-1 in Microglial Cells. Neuron.

[203] Martin M., Michalek S.M., Katz J. (2003). Role of Innate Immune Factors in the Adjuvant Activity of Monophosphoryl Lipid A. Infect. Immun..

[204] Diaz P., Phatak S.S., Xu J., Fronczek F.R., Astruc-Diaz F., Thompson C.M., Cavasotto C.N., Naguib M. (2009). 2, 3-Dihydro-1-Benzofuran Derivatives as a Series of Potent Selective Cannabinoid Receptor 2 Agonists: Design, Synthesis, and Binding Mode Prediction through Ligand-Steered Modeling. ChemMedChem Chem. Enabling Drug Discov..

[205] Xu J.J., Diaz P., Bie B., Astruc-Diaz F., Wu J., Yang H., Brown D.L., Naguib M. (2014). Spinal Gene Expression Profiling and Pathways Analysis of a CB2 Agonist (MDA7)-Targeted Prevention of Paclitaxel-Induced Neuropathy. Neuroscience.

[206] Gong J.-P., Onaivi E.S., Ishiguro H., Liu Q.-R., Tagliaferro P.A., Brusco A., Uhl G.R. (2006). Cannabinoid CB2 Receptors: Immunohistochemical Localization in Rat Brain. Brain Res..

[207] Ashton J.C., Friberg D., Darlington C.L., Smith P.F. (2006). Expression of the Cannabinoid CB2 Receptor in the Rat Cerebellum: An Immunohistochemical Study. Neurosci. Lett..

[208] Beltramo M., Bernardini N., Bertorelli R., Campanella M., Nicolussi E., Fredduzzi S., Reggiani A. (2006). CB2 Receptor-mediated Antihyperalgesia: Possible Direct Involvement of Neural Mechanisms. Eur. J. Neurosci..

[209] Maresz K., Carrier E.J., Ponomarev E.D., Hillard C.J., Dittel B.N. (2005). Modulation of the Cannabinoid CB2 Receptor in Microglial Cells in Response to Inflammatory Stimuli. J. Neurochem..

[210] Van Sickle M.D., Duncan M., Kingsley P.J., Mouihate A., Urbani P., Mackie K., Stella N., Makriyannis A., Piomelli D., Davison J.S. (2005). Identification and functional characterization of brainstem cannabinoid CB2 receptors. Science.

[211] Derbenev A.V., Stuart T.C., Smith B.N. (2004). Cannabinoids Suppress Synaptic Input to Neurones of the Rat Dorsal Motor Nucleus of the Vagus Nerve. J. Physiol..

[212] Pamplona F.A., Prediger R.D.S., Pandolfo P., Takahashi R.N. (2006). The Cannabinoid Receptor Agonist WIN 55,212-2 Facilitates the Extinction of Contextual Fear Memory and Spatial Memory in Rats. Psychopharmacology.

[213] Atwood B.K., Mackie K. (2010). CB2: A Cannabinoid Receptor with an Identity Crisis. Br. J. Pharmacol..

[214] An D., Peigneur S., Hendrickx L.A., Tytgat J. (2020). Targeting Cannabinoid Receptors: Current Status and Prospects of Natural Products. Int. J. Mol. Sci..

[215] Onaivi E.S., Fantini N., Carai M.A.M., Gessa G.L., Colombo G. (2009). CNS Effects of CB2 Cannabinoid Receptors. Open Neuropsychopharmacol. J..

[216] Kivrak B.G., Erzurumlu R.S. (2013). Development of the Principal Nucleus Trigeminal Lemniscal Projections in the Mouse. J. Comp. Neurol..

